# Head and Neck Cancer in Fanconi Anemia: Report of 11 Cases and a Systematic Review

**DOI:** 10.3390/cancers17030349

**Published:** 2025-01-22

**Authors:** Alba de Pablo García-Cuenca, Pau Rofín-Fontanet, Juan Lorente-Guerrero, Judith Balmaña-Gelpí, Estela Carrasco-López, Jordi Temprana-Salvador, Raquel Granado-Carrasco, Irene Braña-García, Pablo Vaquero-Martínez, Rosa Pujol-Pina, Coro Bescós-Atín

**Affiliations:** 1 Service of Oral and Maxillofacial Surgery, Hospital Universitari Vall d’Hebron, 08035 Barcelona, Spain; alba.depablo@vallhebron.cat (A.d.P.G.-C.); pau.rofin@vallhebron.cat (P.R.-F.); pablo.vaquero@vallhebron.cat (P.V.-M.); rosa@rosapujolpina.com (R.P.-P.); 2 Centre d’Investigació en Bioquímica i Biologia Molecular (CIBBIM)-Nanomedicine, New Technologies and Craniofacial Microsurgery, Vall d’Hebron Research Institute (VHIR), Hospital Universitari Vall d’Hebron, 08035 Barcelona, Spain; 3 Service of Otorhinolaryngology, Hospital Universitari Vall d’Hebron, 08035 Barcelona, Spain; juan.lorente@vallhebron.cat; 4 Biomedical Research in Cancer Stem Cells, Vall d’Hebron Research Institute (VHIR), Hospital Universitari Vall d’Hebron, 08035 Barcelona, Spain; 5 Service of Medical Oncology, Hospital Universitari Vall d’Hebron, 08035 Barcelona, Spain; jbalmana@vhio.net (J.B.-G.); ibrana@vhio.net (I.B.-G.); 6 Hereditary Cancer Genetics Group, Vall d’Hebron Institute of Oncology (VHIO), Hospital Universitari Vall d’Hebron, 08035 Barcelona, Spain; ecarrasco@vhio.net; 7 Service of Pathology, Hospital Universitari Vall d’Hebron, 08035 Barcelona, Spain; jordi.temprana@vallhebron.cat; 8 Molecular and Translation Pathology, Vall d’Hebron Research Institute (VHIR), Hospital Universitari Vall d’Hebron, 08035 Barcelona, Spain; 9 Radiation Oncology Group, Vall d’Hebron Institute of Oncology (VHIO), Hospital Universitari Vall d’Hebron, 08035 Barcelona, Spain; raquel.granado@vallhebron.cat; 10 Thoracic Tumors & Head and Neck Cancer Group, Vall d’Hebron Institute of Oncology (VHIO), Hospital Universitari Vall d’Hebron, 08035 Barcelona, Spain; 11 Faculty of Medicine, Universitat Autònoma de Barcelona, 08035 Barcelona, Spain

**Keywords:** head and neck cancer, Fanconi anemia, systematic review, survival, outcome

## Abstract

Background/Objectives: Head and neck cancer is one of the most common malignancies in patients with Fanconi anemia (FA), with a greater than 500-fold incidence of head and neck squamous cell carcinoma. Treatment of HNSCC in these patients is particularly challenging because of poor tolerance to radiation therapy and to some chemotherapeutic agents such as cisplatin. For these reasons, new research is needed to determine the best treatment course for these patients. With this goal in mind, we assessed the characteristics of head and neck squamous cell carcinoma (SCC) in Fanconi anemia (FA). Subjects and Methods: Data for 11 patients (mean age 31 years) with head and neck SCC and FA attended to between 2005 and 2021 were analyzed. Results: The primary tumor site was the oral cavity in eight patients, and five had advanced stages. All patients underwent primary tumor resection. Four patients received adjuvant radiotherapy (mean 57.2 Gy), but three developed toxicity. The mean follow-up was 48.4 months. Six patients experienced 19 episodes of primary tumor recurrence and five developed secondary head and neck tumors. The 3-year disease-free survival was 47%, and the 5-year cause-specific survival was 48%. These findings are similar to the data for 72 patients collected from the literature. Conclusions: The prognosis of head and neck cancer in patients with Fanconi anemia is poor, with an overall survival lower than 50% at 5 years.

## Simple Summary

People with Fanconi anemia (FA) are prone to developing head and neck squamous cell carcinoma (SCC), among other types of cancers. Additionally, they are more prone to suffering cancer relapses and secondary SCC tumors. To make matters worse, they usually suffer serious toxicity-related adverse events when treated with chemotherapy and/or radiotherapy, which sometimes have fatal consequences or force them to stop treatment. For all the reasons mentioned above, the prognosis of SCC in patients with FA is poor, and research and analysis are necessary to determine the best treatment course for these patients, as well as toxicity-related adverse events for each kind of treatment and how to avoid them. The aim of our study is to contribute to the existing knowledge on the subject in order to develop the best possible treatments for FA patients.

## 1. Introduction

Head and neck cancer is one of the most common malignancies in patients with Fanconi anemia (FA), with a greater than 500-fold incidence of head and neck squamous cell carcinoma (HNSCC) relative to the general population [1,2,3]. By 40 years, the cumulative incidence of HNSCC in FA patients is about 14% [3] (Kutler et al., 2003b). This elevated susceptibility arises from a combination of intrinsic genetic abnormalities and external risk factors. FA is characterized by mutations in one of more than 20 genes involved in the repair of interstrand DNA crosslinks. The disruption of the FA/BRCA DNA repair pathway leads to chromosomal instability, accumulation of DNA damage, and increased risk of oncogenic transformation [4,5] (Birkeland et al., 2011; Masserot et al., 2008). Mutations in genes such as FANCA, FANCC, and FANCD2 result in defective homologous recombination, further exacerbating the mutational burden [6,7] (Di Bartolomeo et al., 2022; Tsur et al., 2022). Chronic inflammation and oxidative stress are significant contributors to cancer in FA patients. The presence of reactive oxygen species (ROS) and the inability to repair ROS-induced DNA damage create a microenvironment conducive to malignancy. Additionally, oral epithelial cells in FA patients are particularly vulnerable due to their high turnover rate, leading to a higher likelihood of replication errors [1,8].

Treatment of HNSCC in these patients is particularly challenging because of poor tolerance to radiation therapy [9] and to some chemotherapeutic agents such as cisplatin [10,11]. Radiotherapy may be associated with severe toxicity that requires dose reduction and/or discontinuation of treatment and is known to potentially cause cardiac arrest [1,2] Therefore, it is essential to assess the status of the oral cavity regularly to diagnose oral SCC in early stages, in which only surgical treatment can be a curative option [2,10]. Nevertheless, it is important to consider that local tumor recurrence and development of second new tumors may occur in these patients [1,2]. For all these reasons, the outcome of patients with FA and HNSCC is poor, with a 5-year survival rate lower than 50% [2].

The treatment approach to HNSCC developed for FA patients has not been clearly systematized because of the paucity of data reported in the literature, with most oncological centers having little experience with HNSCC in FA. [11] Lee et al. (2021) performed a systematic review of 119 cases reported from 1996 to 2020 and concluded that surgical resection with curative intent should remain the primary treatment modality; radiation therapy can be administered with acceptable toxicity in most patients; cytotoxic chemotherapy, epidermal growth factor receptor (EGFR) inhibitors, and tyrosine kinase inhibitors may be safe and effective in individual cases, and immunotherapy may also be considered in selected patients. However, the number of published clinical series is limited, and the length of follow-up is relatively short [5,6,7,8]. A total of 35 cases collected from the International Fanconi Anemia Registry (IFAR) represents the largest reported series of HNSCC associated with FA, with the longest follow-up (14 years) based on five patients who were alive at the last follow-up [2].

In this study, we assessed the clinical characteristics and outcomes of 11 patients with HNSCC, with special emphasis on tumor recurrence and second primary tumors. A qualitative systematic review was performed, and data from our patients were compared to those reported in previous case series studies.

Previous research on HNSCC in patients with Fanconi anemia has primarily highlighted the genetic predisposition associated with defects in DNA repair pathways, such as the FA/BRCA pathway. However, many of these studies are constrained by limited sample sizes, variability in methodologies, and insufficient long-term follow-up, leaving critical aspects such as tumor recurrence and the development of secondary malignancies underexplored. In this study, we assessed the clinical characteristics and outcomes of 11 patients with HNSCC, placing special emphasis on tumor recurrence and the development of second primary tumors. Additionally, a qualitative systematic review was performed to compare data from our patients with those reported in previous case series studies, providing a broader context for our findings. This approach not only offers valuable insights into the progression and clinical behavior of these malignancies but also evaluates the efficacy and tolerability of treatment modalities such as surgery, radiotherapy, and chemotherapy to better inform tailored therapeutic strategies for this high-risk population. By synthesizing data from multiple sources and presenting a comprehensive perspective, this work advances the understanding of FA-associated HNSCC and lays the foundation for more standardized approaches to its screening, management, and follow-up.

## 2. Patients and Methods

### 2.1. Study Design and Population

This was a retrospective analysis of prospectively collected data from a cohort of 11 adult patients with FA diagnosed with HNSCC and attended to at the Service of Oral and Maxillofacial Surgery of Hospital Universitari Vall d’Hebron in Barcelona, Spain. Patients were selected based on confirmed diagnoses of FA through chromosomal breakage tests or molecular analysis identifying pathogenic mutations in FA-related genes, as well as histologically confirmed HNSCC located in the oral cavity, oropharynx, larynx, or hypopharynx. To ensure consistency, only patients with comprehensive clinical, pathological, and treatment data were included. Exclusion criteria encompassed individuals with insufficient medical records, incomplete diagnostic confirmation of FA, HNSCC cases outside the specified anatomical regions, or those with less than three months of follow-up. The primary objective of the study was to summarize the clinical characteristics and outcomes of these patients, with a particular focus on tumor recurrence and secondary malignancies. The secondary objective was to perform a systematic review of the literature to compare the findings from our cohort with previously published data on HNSCC in FA patients. The study was approved by the Clinical Research Ethics Committee of Hospital Universitari Vall d’Hebron (code PR(AG)247/2023). Patient consent forms fulfilled all the requisites described in the “Evaluation requirements” established by the Clinical Research Ethics Committee, which include extensive information on patients’ rights and data protection. Data confidentiality was ensured by codification.

### 2.2. Data Collection

For each patient, the following information was recorded: demographics, cancer type, tumor stage according to the 6th edition of the AJCC Cancer Staging Manual [12], histopathologic data, history of hematopoietic stem cell transplantation (HSCT), treatment modality, treatment-related toxicities, local recurrence, second new tumors, follow-up, and outcome. Local recurrence was defined as the presence of a lesion in the same anatomical subsite or adjacent subsite within 3 cm of the primary lesion and developed within a time interval of less than 3 years after the treatment of the primary lesion was completed [13]. A second new tumor was categorized as a head and neck cancer developed in a different location and/or in the same site after more than 3 years of completed treatment of the primary lesion [13]. Outcome variables included overall survival (OS), defined as the time interval from the date of pathological diagnosis to the date of death by any cause or to the last follow-up; cause-specific relative survival (CSRS), defined as the time interval from the date of pathological diagnosis to the date of death due to HNSCC; and disease-free survival (DFS), defined as the time interval from the date of surgery to the date of disease recurrence (recurrence or new HNSCC tumor) or to the last follow-up.

### 2.3. Literature Search and Selection

The Preferred Reporting Items for Systematic Reviews and Meta-Analyses (PRISMA) recommendations were followed [14]. The electronic databases PubMed, Web of Science, Cochrane Library, and Scopus were searched from January 1996 until 30 June 2023. The search terms, either alone or combined, included “Fanconi anemia”, “oral cancer”, “head and neck neoplasms”, “head and neck”, “squamous cell carcinoma”, and “squamous cell carcinoma of head and neck”. The reference lists of all the included articles were thoroughly screened to find other relevant articles. Inclusion criteria were clinical series studies on patients with FA and HNSCC and publication in peer-reviewed English journals. It was also required that the selected studies should include data on clinical characteristics of patients, treatment modality (surgery, radiotherapy, chemotherapy), outcome, and adverse events. Exclusion criteria were publications other than case series (e.g., conference proceedings, reviews, letters, editorials, recommendations, case reports of a single case or two cases); articles published in non-English languages; experimental studies (in vitro, in silico, animal models); studies focused on genetic aspects, risk prediction, and research methodology; esophageal and larynx tumors; oral pre-malignant disorders; hematopoietic stem cell transplant in FA; and articles published in 1995 or in previous years.

After duplicate removals, two reviewers (A.D.P.G-C., P.R.-F.) independently screened all titles and abstracts and then evaluated the full texts of the eligible articles based on the inclusion criteria, and any disagreement was resolved by discussion. Extracted data, including article title and authors, hospital/healthcare center where the study was performed, publication date, number of patients included in the series, patient demographics, type of cancer, tumor-related data, histopathology data, modality of treatment (surgery, chemotherapy, radiotherapy), treatment-associated toxicities, recurrence (local/regional), follow-up, and outcome were collected in an electronic database.

**Figure 1:**
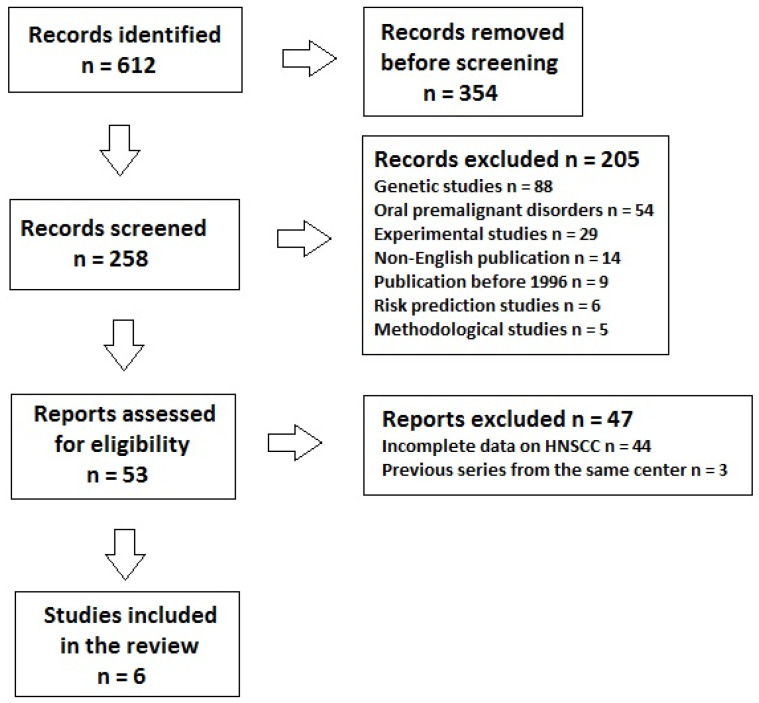
Preferred Reporting Items for Systematic Reviews and Meta-Analyses (PRISMA) flow diagram for the systematic literature review depicting the number of records identified, included, and excluded, along with the reasons for exclusions.

A total of 612 articles were retrieved, 354 of which were removed before screening (e.g., conference proceedings, case reports, consensus, recommendations, etc.). Of the remaining 258 articles, 205 did not meet the inclusion criteria and were excluded. Fifty-three full-text articles were assessed and 47 were excluded because of incomplete information on HNSCC or previous series from the same center; therefore, a total of 6 studies were analyzed (Figure 1).

### 2.4. Statistical Analysis

The Kaplan–Meier method was used to assess OS, CSRS, and DRFS. The probabilities of survival were calculated at follow-up time points of 1, 3, and 5 years. Median survival was also estimated, with the 50th percentile of the distributions. For DFS, two analyses were performed considering (a) recurrence of the primary tumor or development of a new head and neck malignant lesion as the event of interest, censoring disease-free patients, and (b) recurrence of the primary tumor as the event of interest, censoring disease-free patients and those who had developed a second new head and neck tumor at follow-up. Differences between survival curves were analyzed with the log-rank test. Statistical significance was set at *p* < 0.05. Descriptive statistics were used to summarize the results of the systematic review.

## 3. Results

### 3.1. Study Population

A total of 11 patients—six men and five women—with a mean age of 31 years (range 25–42 years) presented with FA and HNSCC during the study period. The diagnosis of Fanconi anemia was confirmed by *FANCA* gene mutation in 10 patients and *FANCD2* mutation in one. The clinical characteristics of the patients are shown in Table 1. All tumors were squamous cell carcinomas and affected the oral cavity in eight cases and the oropharynx, larynx, and hypopharynx in one case each. One patient developed two synchronous tumors affecting the retromolar trigone and the hard palate. The tongue and the retromolar trigone were the most common tumor locations. Advanced stages (III–IV) were diagnosed in five patients, whereas the remaining six had stages 0–II. Five patients had received HSCT. Also, premalignant lesions had been diagnosed in nine patients.

As shown in Table 1, surgical resection of the tumor was accomplished in all patients and associated with cervical neck dissection in six patients. Reconstruction of the defect after resection was performed in four patients using microsurgery; an anterolateral thigh microsurgical flap was used in two patients, a sural flap in one, and a fibula flap in one. The four patients undergoing microsurgical reconstruction required blood transfusion. Two patients (18.2%) developed postoperative complications, including a neck infection in one and a cervical chyloma and pneumonia in the other. Histopathological examination of the surgical specimen revealed free margins in three patients, positive margins in three, and margins close to the malignant lesion in five.

Three patients received adjuvant postoperative radiotherapy, and one received concurrent chemoradiotherapy. The radiotherapy modality was 3D conformal in one patient and intensity-modulated radiation therapy in three. The mean dose was 57.2 Gy (range 46.8–66 Gy). Toxicity (mucositis, epithelitis, and odynophagia) was the reason for the transient interruption of radiation therapy in two patients and permanent discontinuation in one patient. In the patient treated with concurrent chemoradiotherapy, chemotherapy was withdrawn due to grade 3 mucositis after one cycle of cisplatin (low-dose cisplatin 30 mg/m^2^ due to renal clearance lower than 60 mL/min).

### 3.2. Recurrence and Second New Tumors

Recurrence of the primary lesions occurred in six patients (54.5%), with a total of 19 episodes of recurrence (local recurrence in 17, locoregional with metastasis in 2). Second new tumors were diagnosed in five (45.4%) patients, involving the alveolar ridge in two, the oral tongue in one, the labial mucosa in one, and the palate in one. Stages included Tis (in situ) in one case, stage I in one case, and stage II in two cases. In one case, the stage was unknown. Of the 24 episodes of recurrence (including the five patients with second tumors), 20 (83.3%) were treated with surgery (local resection in 18, locoregional resection in 2) and the remaining four with palliative care.

### 3.3. Outcomes

After a mean follow-up of 48.4 months (range 8–108; median 58 months), five patients were alive; four of them showed no evidence of disease. The remaining six patients died. Causes of death were progression of HNSCC in four patients (recurrence in three, new second tumor in one), hematologic disease in one patient, and breast cancer in one patient.

**Figure 2:**
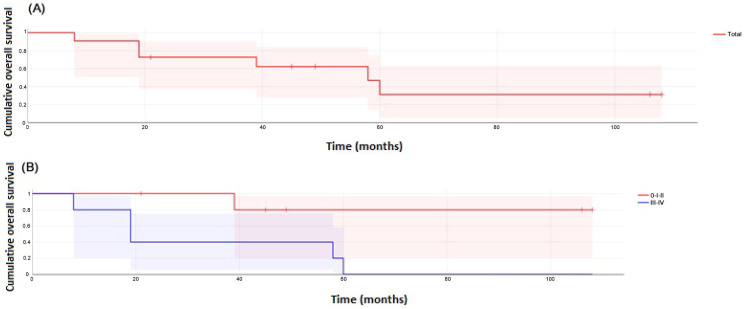
(**A**) Overall survival of 11 patients with FA and HNSCC. (**B**) Overall survival curve of 6 FA patients with HNSCC stages 0–II and of 5 FA patients with HNSCC stages III–IV (log-rank test, *p* = 0.028).

**Figure 3:**
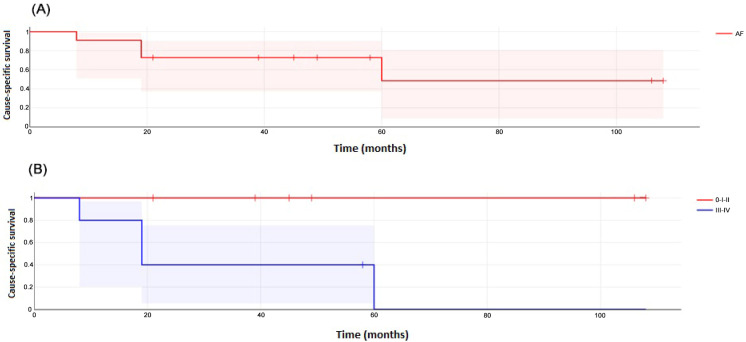
(**A**) Cause-specific relative survival of 11 patients with FA and HNSCC. (**B**) Cause-specific relative survival of 6 FA patients with HNSCC stages 0–II and of 6 patients with HNSCC stages III–IV (log-rank test, *p* = 0.013).

The OS was 90% at 1 year, 73% at 3 years, and 47% at 5 years (Figure 2A). There were statistically significant differences in OS between patients with tumor stages I–II and those with stages III–IV (*p* = 0.028) (Figure 2B). The CSRS was 91% at 1 year, 73% at 3 years, and 48% at 5 years (Figure 3A). Also, there were significant differences according to tumor stage (*p* = 0.013) (Figure 3B). Other differences in OS and CSRS rates according to age, treatment modality, status of surgical margins, or previous HSCT were not found.

**Figure 4:**
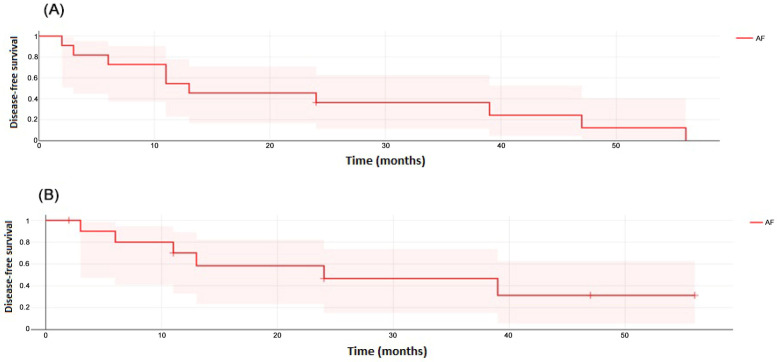
(**A**) Disease-free survival of 11 patients with FA and HNSCC when considering patients without recurrence of the primary tumor and without the development of a second new head and neck tumor. (**B**) Disease-free survival when considering patients without recurrence of the primary tumor only.

The mean DFS was 21.4 months (range 3–56 months). The DFS was 55% at 1 year and 36% at 3 years when considering patients without recurrence of the primary tumor and without the development of a second new head and neck tumor (Figure 4A), whereas DFS was 70% at 1 year and 47% at 3 years when considering patients without recurrence of the primary tumor only (Figure 4B).

### 3.4. Literature Review

The six studies included data from 72 patients with HNSCC and FA [2,5,6,7,8,9]. The descriptive characteristics of these 72 patients and our series of 11 patients are shown in Table 2 and Table 3.

## 4. Discussion

Head and neck cancer is a frequent malignancy developed in patients with FA. Patients with Fanconi anemia have a much higher probability of developing malignancies from an early age, and such patients are 50 times more likely to develop solid tumors (including liver, brain, renal, and genital tumors, among others) than people without FA [3,5]. Leukemia is one of the most common malignancies developed by FA patients, which is particularly troubling because HSCT (frequently used as a treatment against leukemia) is known to increase the risk of oral squamous cell carcinoma (OSSC) [15]. In a comparison of two cohorts of FA patients, 145 patients who did not receive transplants and 117 who received transplants, the age-specific hazard of HNSCC was 4.4-fold higher in patients who received transplants than in those who did not, and malignancy occurred at significantly younger ages [16]. In the present review, a history of HSCT was registered in more than half of patients, with a rate of 56.6%.

Another consistent finding was the young age of FA patients with HNSCC: a mean of 26.3 years in the studies collected from the literature and 31 years in our series, as compared with people without FA in which HNSCC is more prevalent after the age of 50–60 years [17]. Moreover, more than 70% of patients presented with HNSCC of the oral cavity, with the tongue being the most frequent tumor location. In the study by Di Bartolomeo et al. [6], all cases were found in the tongue, which has been explained by the high turnover of mucosal cells of the dorsal surface due to a continuous movement stimulus with a replicative error more frequent in FA. Repairing DNA damage due to traumatic friction also causes a potential increase in replicative error [6]. In our study, however, the most frequent tumor site was the retromolar trigone in the oral cavity, which affected 27% (3/11) of the patients, followed by the oral tongue in 18% of patients (2/11). The retromolar trigone is another frequent location of HNSCCs developed by FA patients, as has been reported in the series by Anak et al. [8], where 100% of the tumors were found in the retromolar trigone.

Premalignant lesions of the oral cavity may be the reason for close surveillance of patients, which may result in a diagnosis of cancer at early stages. On the other hand, screening for malignancies in patients diagnosed with FA also contributes to the early detection of HNSCC. Unfortunately, many tumors are diagnosed at advanced (III and IV) stages (between 45% and 75% in the present review).

Surgery of the primary tumor is the treatment of choice, which was performed in 100% of our patients and in 80% of the patients reported in the different series [2,7,8,9]. Neck dissection was performed in 6 of our 11 patients, with a rate of 54.5%, which was somewhat lower than the 63% reported by Kutler et al [2].Elective neck dissection in early-stage HNSCC in non-FA patients remains a matter of debate, and depth of invasion ≥ 4 mm has been proposed as a key parameter to perform elective neck dissection in early-stage oral cancer [18]. Postoperative complications have been reported in 23% of patients, with hardware-related wound infection as one of the most common. Plate-related complications leading to plate exposure have been shown to be a frequent cause of surgical site infections following oral cavity cancer resection and reconstruction [19]. In the series by Kutler et al. [2], hardware removal was necessary in two patients with wound infection. However, extensive resection of primary head and neck tumors is feasible in FA patients.

Standard treatment of HNSCC incorporates surgery in combination with radiotherapy and chemotherapy, depending on tumor characteristics; however, sensitivity to radiation therapy and chemotherapy agents (e.g., cisplatin) may limit the use of these treatments in FA patients. In our study, 4 of the 11 patients (36.4%) received radiotherapy, which is somewhat lower than the 30 of 72 patients (41.7%) reported in the other series [2,5,6,8,9]. Radiotherapy was mostly administered in the adjuvant setting; however, radiotherapy was interrupted in three of our patients (75%) as compared to 11 of 30 patients (36.7%) reported in the reviewed studies [2,5,8,9], probably because of the highest mean dose (57.2 vs. 47 Gy) used in our study. In the assessment of individual studies, a very high rate of radiotherapy discontinuation—67% and 100%—was found in two studies [2,8] despite the fact that they used very low mean doses—34.3 and 36.2 Gy, respectively—whereas no interruptions were reported in another study using a high mean dose of 68.6 Gy [7].

Some of the most prevalent complications during radiation therapy are high-grade mucositis, hematologic abnormalities (pancytopenia), and dysphagia [2,5,8,9]. Other complications reported include asystole with cardiac arrest, wound site breakdown, carotid bleeds, fibrosis, local edema, sepsis, tracheal stenosis, and radiation pneumonitis [2,9]. In our study, the reasons for radiation therapy discontinuation were severe mucositis, epithelitis, and odynophagia, although hematological or severe life-threatening complications were not observed. Significant toxicities may be reduced through lower daily doses delivered over longer courses, although mucositis and blood cell counts should be monitored closely [4].

Chemotherapy was used in a few patients, except in the series of nine patients reported by Beckham et al. [9], in which five (56%) of the patients received different regimens based on cetuximab, carboplatin, paclitaxel, nivolumab, and tremelimumab/durvalumab. In our study, one patient received low-dose cisplatin, but chemotherapy had to be stopped after 1 cycle due to grade 3 mucositis. Cytotoxic chemotherapy (usually with a platinum-based regimen), either with or without radiotherapy, is not well tolerated by most FA patients with HNSCC [11]. In the present review, seven patients received cetuximab in addition to adjuvant or neoadjuvant radiotherapy [2,9] and complications were reported in two of them, including high-grade mucositis, dysphagia, cytopenia, and brisk dermatitis in the radiation field, although the extent to which these effects were related to cetuximab, radiation, or a combination of the two remained unclear.

Local recurrence of the primary lesion and rapid progression is common in patients with FA and HNSCC, even during treatment or shortly after completion of therapy. A recurrence rate of 54% was observed in our study and also in the overall recurrence rate of the series analyzed [2,5,6,7,8,9]. The median DFS of 21.4 months was also similar to the 22 months found in 35 patients reported by Kutler et al. [2]. Also, DFS decreased from 70% at 1 year to 47% at 3 years in our study, which is similar to the 2-year and 3-year DFS of 75% and 43% shown by Kutler et al. [2]. It should be noted that, when considering not only the recurrence but also the second head and neck tumor, DFS decreased to 55% (1 year) and 36% (3 years), mostly because second new tumors developed in 4 of our 11 patients (36.4%). Recurrences of primary lesions, second new tumors, and treatment-related toxicities account for the low OS rate, which was 47% at 5 years in our study as compared with 39% in the study by Kutler et al. [2]. However, the 5-year CSRS in both studies was very similar: 45% in the series by Kutler et al. [2] and 48% in the present study.

### Limitations of Our Study

This study highlights important findings about the occurrence and management of head and neck squamous cell carcinoma (HNSCC) in patients with Fanconi anemia (FA), but certain limitations must be addressed. The rarity of FA and associated malignancies inherently limits the sample size, reducing the generalizability of the results. The retrospective nature of the data introduces potential biases and heterogeneity in diagnostic, treatment, and follow-up protocols. Additionally, incomplete molecular and genetic data across cases restricts the ability to fully explore the relationship between specific FA mutations and cancer risk. Variability in treatment approaches—including differences in surgical techniques, radiotherapy protocols, and chemotherapy regimens—hampers the direct comparison of outcomes. Follow-up duration was inconsistent, with some patients lost to follow-up, which affected the reliability of survival and recurrence analyses. Furthermore, the impact of hematopoietic stem cell transplantation (HSCT), a known risk factor for secondary malignancies in FA, is difficult to disentangle due to variations in conditioning regimens and the presence of chronic graft-versus-host disease. Despite these limitations, this study underscores the need for standardized screening and treatment strategies, as well as prospective, multi-center research to improve outcomes in this high-risk population.

## 5. Future Perspectives

Further studies are essential to optimize oncologic outcomes in FA patients with HNSCC, in particular, to define screening programs for diagnosing the disease in the earliest possible stages. Due to the burden of multiple biopsies performed during regular screening, future directions on non-invasive screening methods, such as brush cytology [20] or liquid biopsy [21], are of utmost interest. Also, studies focused on chemoprophylaxis of oral cancer and management of oral premalignant disorders in this population are necessary. Other lines of research involve the characterization of alternative radiotherapy protocols, the use of less aggressive cytotoxic agents, and improvement in our experience with EGFR agents [22] and immune checkpoint inhibitors.

## 6. Conclusions

Despite limitations in the current evidence based on a clinical series of 72 patients with FA and HNSCC to which 11 cases from our experience were added, surgical resection of the primary tumor—in most cases affecting the tongue and the retromolar trigone—was the standard treatment approach, which was combined with radiation therapy (usually in the adjuvant setting) in 41% of cases. History of HSCT was recorded in 78% of cases, and about half of the patients had an advanced stage (III–IV) at presentation. Radiotherapy had to be interrupted in 45% of patients due to toxicities, in some cases, despite using very low mean radiation doses (34.3 Gy). The overall recurrence rate of the primary tumor was high (54%), and a second new head and neck tumor occurred in 45.4% of our series. The outcome of FA patients with HNSCC is poor, with a median DFS of around 21 months and OS of 47% at 5 years.

These findings underscore the significant clinical burden of HNSCC in FA patients and highlight the need for tailored screening programs and individualized therapeutic strategies to improve outcomes. This is particularly relevant given the high recurrence rate and low survival rate of patients with FA and HNSCC. Future research should prioritize genomic studies to identify molecular markers predictive of treatment response, recurrence risk, and long-term outcomes. Such studies could enhance the precision of therapeutic strategies and pave the way for the development of targeted therapies that address the unique genetic and molecular landscape of FA-associated HNSCC. Additionally, multicenter prospective studies with standardized treatment protocols are essential to refine current management approaches and establish evidence-based guidelines for this high-risk group. By addressing these critical gaps, future efforts can contribute to improving survival and quality of life for patients with FA and HNSCC.

## Figures and Tables

**Table 1:** Clinical characteristics and treatments of patients with HNSCC and FA.

Case	Age at HNSCC Diagnosis Years	Tumor Location	TNM	Stage	Treatment	Surgical Margins	Radiotherapy Modality (Total Dose)	Toxicities	Adjuvant Treatment
1	25	Oral tongue	T2N1M0	III	Surgery LR + adjuvant RT	Close	3D conformal (46.8 Gy)	G3 mucositis G2 epithelitis G2 odynophagia	RT completed with transient interruption
2	33	Base tongue	T4aN1M0	IVa	Surgery LR + ALT	Free			
3	34	Oral tongue	T1N0M0	I	Surgery L	Free			
4	27	Lateral pharynx	T1N0M0	I	Surgery L	Positive			
5	30	Hard palate	T1N0M0	I	Surgery L	Positive			
6	23	Retromolar trigone	T1N0M0	I	Surgery L	Close			
7	33	Vocal cord	TisN0M0	0	Surgery L	Free			
8	30	Alveolar ridge	T4aN1M0	IVa	Surgery LR, ALT + CCRT	Positive	IMRT (66 Gy)	G3 mucositis	Chemotherapy stopped after 1 cycle
9	42	Retromolar trigone + hard palate	T4aN1M0 T1	IVa I	Surgery LR, FFF + adjuvant RT	Close	IMRT (56 Gy)	G3 odynophagia	RT completed with transient interruption
10	31	Retromolar trigone	T4aN3aM0	IVa	Surgery LR + adjuvant RT	Close	IMRT (56 Gy)	G3 mucositis G3 epithelitis	RT stopped
11	33	Hard palate	T2N0M0	II	Surgery LR, SFF + adjuvant RT	Close			

HNSCC: head and neck squamous cell carcinoma; FA: Fanconi anemia; LR: locoregional resection; RT: radiotherapy; ALT: anterolateral thigh free flap; L: local resection; CCRT: concurrent chemoradiotherapy; IMRT: intensity modulated radiation therapy; FFF: free fibula flap; SFF: sural free flap; G: grade.

**Table 2:** Salient data of 83 patients with HNSCC and FA.

First Author Year	Patients (Male/ Female)	Age, Years Mean (Range)	HSCT	HNSCC Oral Cavity	Stages III–IV	Surgery	Radiation Therapy	Dose (Gy) Mean (Range)	Radiation Therapy Interruption	Chemotherapy
Masserot et al. [5]	13 (9/4)	20.6 (10–32.6)	13 (100)	11 (85%)	6 (46%)	10 (77%)	3 (23%) Adjuvant 1 Neoadjuvant 2	34.3 (22–70)	2/3 (67%)	1 (7%)
Kutler et al. [2]	35 (13/22)	32 (14–48)	13 (37%)	26 (74%)	16 (46%)	30 (86%)	16 (46%) Adjuvant 13 Neoadjuvant 1 Palliative 3	50.5 (25–70.2)	5/16 (31%)	3 (9%) Cetuximab 3
Beckham et al. [9]	9 (6/3)	33.4	5 (56%)	7 (78%)	5 (56%)	8 (89%)	5 (56%) Adjuvant 5 (Proton 1)	45.5 (3.7–70)	1/4 (25%) Unavailable 1	5 (56%) Cetuximab 4 Carboplatin-paclitaxel 1 Paclitaxel 1 Nivolumab 1 Tremelimumab/durvalumab 1
Anak et al. [8]	4 (3/1)	21 (15–32)	4 (100%)	4 (100%)	3 (75%)	3 (75%)	3 (75%) Adjuvant 2 Palliative 1	36.2 (32.3–45.9	3/3 (100%)	3 (75%)
Di Bartolomeo et al. [6]	5 (2/3)	21.7 (18.4–28.8)	5 (100%)	5 (100%)	3 (60%)	3 (60%)				3 (60%)
Tsur et al. [7]	6 (2/4)	29 (15–49)	2 (33%)	4 (67%)	3 (50%)	4 (67%)	3 (50%) Adjuvant 2 Neoadjuvant 1	68.6 (66–70)	0/3	3 (50%)
de Pablo García-Cuenca et al. [Present study]	11 (4/7)	31 (25–42)	5 (45%)	8 (73%)	5 (45%)	11 (100%)	4 (36%) Adjuvant 4	57.2 (46.8–66)	3/4 (75%)	1 (9%)

HNSCC: head and neck squamous cell carcinoma; FA: Fanconi anemia; HSCT: hematopoietic stem cell transplantation.

**Table 3:** Outcome of 83 patients with HNSCC and FA.

First Author Year	Patients	Recurrence Primary Tumor	Overall Survival	Disease-Free Survival	Cause-Specific Relative Survival
Masserot et al. [5]	13	10/13 (77%)	15.4% (2/13) 2 alive at 9 and 23 months 11 died (2.5 to 46.5 months)		
Kutler et al. [2]	35	17/35 (49%)	14.3% (5/35) 5 alive, 30 died 2-year 71% 5-year 39% Median 42 months	2-year 75% 5-year 43% Median 22 months	2-year 79% 5-year 47%
Beckham et al. [9]	9	3/9 (33%)	33.3% (3/9) 3 alive after 8, 21, and 26 months, respectively 6 died (7 weeks to 20 months)		
Anak et al. [8]	4	3/4 (75%)	0% (4/4) 4 died, median 6 months (6 to 12 months)		
Di Bartolomeo et al. [6]	5	4/5 (80%)	20% (1/5) 1 alive after 7 months 4 died (2 to 13 months)		
Tsur et al. [7]	6	2/6 (33%)	Median 11 months (0.5 to 57 months)		
de Pablo García-Cuenca et al. [Present study]	11	6/11 (54%)	45.4% (5/11) 5 alive, 6 died 1-year 90% 3-year 73% 5-year 47% Median 58 months	1-year 70% 3-year 47% Median 21.4 months	1-year 91% 3-year 73% 5-year 48%

HNSCC: head and neck squamous cell carcinoma; FA: Fanconi anemia.

## Data Availability

The data that support the findings of this study are available from the first author upon request.

## References

[1] Kutler D.I., Auerbach A.D., Satagopan J., Giampietro P.F., Batish S.D., Huvos A.G., Goberdhan A., Shah J.P., Singh B. (2003). High incidence of head and neck squamous cell carcinoma in patients with Fanconi anemia. Arch. Otolaryngol. Head Neck Surg..

[2] Kutler D.I., Patel K.R., Auerbach A.D., Giampietro P.F., Batish S.D., Huvos A.G., Goberdhan A., Shah J.P., Singh B. (2016). Natural history and management of Fanconi anemia patients with head and neck cancer: A 10-year follow-up. Laryngoscope.

[3] Kutler D.I., Singh B., Satagopan J., Batish S.D., Berwick M., Giampietro P.F., Hanenberg H., Auerbach A.D. (2003). A 20-year perspective on the International Fanconi Anemia Registry (IFAR). Blood.

[4] Birkeland A.C., Auerbach A.D., Sanborn E., Parashar B., Kuhel W.I., Chandrasekharappa S.C., Smogorzewska A., Kutler D.I. (2011). Postoperative clinical radiosensitivity in patients with Fanconi anemia and head and neck squamous cell carcinoma. Arch. Otolaryngol. Head Neck Surg..

[5] Masserot C., Peffault de Latour R., Rocha V., Leblanc T., Rigolet A., Pascal F., Janin A., Soulier J., Gluckman E., Socié G. (2008). Head and neck squamous cell carcinoma in 13 patients with Fanconi anemia after hematopoietic stem cell transplantation. Cancer.

[6] Di Bartolomeo M., Anesi A., Pellacani A., Negrello S., Natale A., Figurelli S., Vaddinelli D., Angelini S., Chiarini L., Nocini R. (2022). Tongue cancer following hematopoietic cell transplantation for Fanconi anemia. Clin. Oral Investig..

[7] Tsur N., Frig O., Steinberg-Shemer O., Kurman N., Mizrachi A., Popovtzer A. (2022). Characterization of Fanconi anemia patients with head and neck squamous cell carcinoma: Israel Fanconi Registry. Isr. Med. Assoc. J..

[8] Anak S., Yalman N., Bilgen H., Sepet E., Deviren A., Gürtekin B., Tunca F., Başaran B. (2020). Squamous cell carcinoma development in Fanconi anemia patients who underwent hematopoietic stem cell transplantation. Pediatr. Transplant..

[9] Beckham T.H., Leeman J., Jillian T.C., Riaz N., Sherman E., Singh B., Lee N., McBride S., Higginson D.S. (2019). Treatment modalities and outcomes of Fanconi anemia patients with head and neck squamous cell carcinoma: Series of 9 cases and review of the literature. Head Neck.

[10] Amenábar J.M., Torres-Pereira C.C., Tang K.D., Punyadeera C. (2019). Two enemies, one fight: An update of oral cancer in patients with Fanconi anemia. Cancer.

[11] Lee R.H., Kang H., Yom S.S., Smogorzewska A., Johnson D.E., Grandis J.R. (2021). Treatment of Fanconi anemia-associated head and neck cancer: Opportunities to improve outcomes. Clin. Cancer Res..

[12] Green F.L., Page D.L., Fritz A., Balch C.M., Haller D.G., Morrow M. (2002). AJCC Cancer Staging Manual.

[13] Rohde M., Rosenberg T., Pareek M., Nankivell P., Sharma N., Mehanna H., Godballe C. (2020). Definition of locally recurrent head and neck squamous cell carcinoma: A systematic review and proposal for the Odense-Birmingham definition. Eur. Arch. Otorhinolaryngol..

[14] Liberati A., Altman D.G., Tetzlaff J., Mulrow C., Gøtzsche P.C., Ioannidis J.P., Clarke M., Devereaux P.J., Kleijnen J., Moher D. (2009). The PRISMA statement for reporting systematic reviews and meta-analyses of studies that evaluate healthcare interventions: Explanation and elaboration. BMJ.

[15] Murillo-Sanjuán L., Balmaña J., de Pablo García-Cuenca A., Lorente Guerrero J., Uria Oficialdegui M.L., Carrasco E., Diaz-de-Heredia C. (2022). Post-hematopoietic stem cell transplant squamous cell carcinoma in patients with Fanconi anemia: A dreadful enemy. Clin. Transl. Oncol..

[16] Rosenberg P.S., Socié G., Alter B.P., Gluckman E. (2005). Risk of head and neck squamous cell cancer and death in patients with Fanconi anemia who did and did not receive transplants. Blood.

[17] Xu Q., Wang C., Li B., Kim K., Li J., Mao M., Qin L., Li H., Huang X., Xing R. (2019). The impact of age on oral squamous cell carcinoma: A longitudinal cohort study of 2,782 patients. Oral Dis..

[18] van Lanschot C.G.F., Klazen Y.P., de Ridder M.A.J., Mast H., Ten Hove I., Hardillo J.A., Monserez D.A., Sewnaik A., Meeuwis C.A., Keereweer S. (2020). Depth of invasion in early stage oral cavity squamous cell carcinoma: The optimal cut-off value for elective neck dissection. Oral Oncol..

[19] Yao C.M., Ziai H., Tsang G., Brown D., Irish J.C., Gilbert R.W., Goldstein D.P., Gullane P.J., de Almeida J.R. (2017). Surgical site infections following oral cavity cancer resection and reconstruction is a risk factor for plate exposure. J. Otolaryngol. Head Neck Surg..

[20] Velleuer E., Dietrich R., Pomjanski N., Araujo I.K., Baretton G.B., Maier H., Dietz A., Meerpohl J.J., Lohmann D., Digweed M. (2010). Diagnostic accuracy of brush biopsy-based cytology for the early detection of oral cancer and precursors in Fanconi anemia. Cancer Cytopathol..

[21] Errazquin R., Carrasco E., Del Marro S., Suñol A., Peral J., Ortiz J., Rubio J.C., Segrelles C., Dueñas M., Garrido-Aranda A. (2023). Early diagnosis of oral cancer and lesions in Fanconi anemia patients: A prospective and longitudinal study using saliva and plasma. Cancers.

[22] Montanuy H., Martínez-Barriocanal Á., Casado J.A., Rovirosa L., Ramírez M.J., Nieto R., Carrascoso-Rubio C., Riera P., González A., Lerma E. (2020). Gefitinib and afatinib show potential efficacy for Fanconi anemia-related head and neck cancer. Clin. Cancer Res..

